# The impact of the expanded health insurance coverage policy on healthcare spending: evidence from Korea

**DOI:** 10.1186/s12939-024-02206-3

**Published:** 2024-06-21

**Authors:** Seokmin Ji, Munjae Lee, Mankyu Choi, Sewon Park

**Affiliations:** 1 Department of Health Policy & Management, College of Health Science, Korea University, Seoul, South Korea; 2 BK21 FOUR R&E Center for Learning Health Systems, Korea University, Seoul, South Korea; 3 Department of Medical Science, Ajou University School of Medicine, Suwon, 16499 South Korea

**Keywords:** National health insurance (NHI), Health insurance coverage, Health expenditures, Difference-in-differences, Policy evaluation

## Abstract

**Background:**

South Korea’s National Health Insurance (NHI) system pursues universal health coverage, but it has not been able to alleviate patients’ financial burden owing to limited coverage and a high proportion of out-of-pocket expenses. In 2017, the government announced a plan to strengthen universality by providing coverage for all unincluded services, expanding coverage, and alleviating household financial burden. We aimed to evaluate the effect of “Moon Care” with a focus on changes in health expenditures following policy implementation, and to provide empirical evidence for future policies to strengthen the NHI system’s universality.

**Methods:**

Using data from the 2016 and 2018 Korea Health Panel (KHP), we established a treatment group affected by the policy and an unaffected control group; we ensured homogeneity between the groups using propensity score matching (PSM). Subsequently, we examined changes in NHI payments, non-payments, and out-of-pocket payments (OOP); we performed difference-in-differences (DID) analysis to evaluate the policy’s effect.

**Results:**

Following policy implementation, the control group had a higher increase than the treatment group in all categories of health expenditures, including NHI payments, non-payments, and OOP. We noted significant decreases in all three categories of health expenditures when comparing the differences before and after policy implementation, as well as between the treatment and control groups. However, we witnessed a significant decrease in the interaction term, which confirms the policy’s effect, but only for non-payments.

**Conclusions:**

We observed the policy’s intervention effect over time as a decrease in non-payments, on the effectivity of remunerating covered medical services. However, the policy did not work for NHI payments and OOP, suggesting that it failed to control the creation of new non-covered services as noncovered services were converted into covered ones. Thus, it is crucial to discuss the financial spending of health insurance regarding the inclusion of non-covered services in the NHI benefits package.

## Background

Universal Health Coverage (UHC) refers to the constitution of a sustainable healthcare system to ensure that everyone can receive medical services without financial hardship [1]. The National Health Insurance (NHI) helps to ease the financial burden of unexpected medical costs on individuals and families, but in the case of the US, where there is no NHI, people sometimes go bankrupt because of their medical costs; their lowered accessibility to treatments for diseases may also pose a threat to health [2, 3]. When medical costs are not covered by health insurance, patients face financial difficulties or discontinue treatment. In addition, the expansion of health insurance coverage is becoming increasingly important because of the increase in medical costs due to an aging population.

The government suggested a roadmap to reinforce health insurance coverage in 2005 and then established and implemented mid- to long-term plans every 4–5 years, regardless of the nature of the government, for policy continuity. Examining this by period, the goal of the 1st Reinforcement Plan of NHI Coverage (2005–2008) was to drastically reduce the ratio of patient burden centered on severe diseases with high medical cost burdens. Non-payment items for severe diseases were converted into benefit items, and legal out-of-pocket payments (OOP) were reduced from 20 to 10%. In the 2nd Reinforcement Plan of NHI Coverage (2009–2013), to alleviate the burden of medical costs for severely ill and high-cost patients, the scope of insurance coverage for the treatment of severe diseases was expanded, and the OOP rate for cancer and cardiac disorders was reduced from 10 to 5%. In addition, among the non-payment items, magnetic resonance imaging (MRI) scans for spinal and joint diseases were converted into benefits, and the inclusion of ultrasonography into insurance coverage was promoted. In the 3rd Reinforcement Plan of NHI Coverage (2014–2018), to ensure essential medical care for health problems by life cycle, medical support for pregnancy, childbirth, and newborns was expanded, and support was strengthened for early management of key ailments in adolescents, young adults, and middle-aged people. Furthermore, expensive non-payment items were alleviated, health insurance coverage was expanded for high-priced MRI scans and ultrasonography, and health insurance support was strengthened not only for undeserved brackets, such as the disabled and low-income populations, but also for vulnerable areas of essential medical care [4, 5].

Despite government efforts, there was a limit to improving the health insurance coverage rate owing to the severe disease-oriented coverage expansion. Accordingly, the increase in co-payment for non-payment items was regarded as a factor impeding the improvement of the health insurance coverage rate; therefore, from the 3rd Reinforcement Plan of NHI Coverage onward, non-payment medical services were converted into covered ones. In this regard, the Moon Jae-in administration succeeded at enhancing previous governments’ efforts to expand coverage and announced “Moon Care,” a reinforcement measure of NHI coverage (2017–2022), in 2017. “Moon Care” aims to reduce the co-payment rate for medical costs by expanding the health insurance coverage rate up to 70% in order to prevent people from descending into poverty due to medical expenses. Targeting the “coverage of all non-payment medical items,” it was promoted in three directions: (1) converting non-payment items into payment items; (2) easing the burden of medical expenses for vulnerable citizens; and (3) reinforcing the medical safety net, through which it intended to drastically lower medical care expenses for the general population.

Examining this in detail, “Moon Care” intended to incorporate all non-payment medical items into health insurance and to abolish the three major non-payment items. Through a preliminary payment system that differentially applies out-of-pocket payments (OOP) rates of 30–90% to 3,800 non-payment items required for treatment, it intended to gradually incorporate them into benefits. In particular, non-payment items required for exams and treatments, such as ultrasonography and magnetic resonance imaging (MRI), were gradually incorporated into health insurance. Furthermore, it was intended to ease the burden of medical expenses by abolishing the three major non-payment items of optional medical costs, advanced hospital room fees, and nursing fees, which accounted for 60% of non-payment costs [6–8].

For “Moon Care,” there are positive and negative views at the same time. On the one hand, “Moon Care” has eased the burden of national medical costs through the expansion of coverage. On the other hand, “Moon Care” causes wasteful spending due to the increase in covered non-payment treatments [5, 9]. In this situation, given that the ultimate goal of ‘Moon Care’ is to reduce the burden of medical expenses on households, it is necessary to monitor and objectively evaluate how the actual burden of medical expenses and the level of access to necessary services have changed since the introduction of the policy. In addition, monitoring should also be done on how the national financial resources used to achieve the goal of reducing the burden of medical expenses on households are changing together.

There are three types of medical expenses incurred in the Korean healthcare system: NHI payments, OOP, and non-payments, and their sum represents the total medical expenses calculated at the national level. These are calculated as indicators such as current health expenditures, catastrophic medical expenditures, public financial contribution rate, and health insurance coverage rate, and are used as key variables to set the direction of the national healthcare system and evaluate its performance [9]. Utilizing this, we investigated the changes in the disbursement of medical expenses by utilizing national data through which we were able to identify the changes in medical expenses before (2016) and after the policy (2018), and then to verify the policy’s effectiveness. We provide basic data for future health insurance coverage policies and present policy implications for achieving UHC.

## Methods

### Data sources

We used Korean Health Panel (KHP) data to measure the effectiveness of the Reinforcement Plan of NHI Coverage, which was implemented in 2017. The KHP uses the enumeration district of the 2005 Population and Housing Census as a sampling frame and thus has national-level representativeness; it is characteristic of panel data to provide medical expenditure amounts, medical usage (outpatient, hospitalization, and emergency), health behavior, and sociodemographic information, among other aspects. As these data are surveyed for the same households and household members every year, it is possible to analyze changes in medical expenditures at the household or individual level according to the policy [10]. Annual data from 2016, before the policy was enforced, and from 2018 were combined to confirm changes in NHI payments, non-payment items, and OOP. The data are available from the authors upon reasonable request and with the permission of the Korea Institute for Health and Social Affairs and the National Health Insurance Service. All original data are publicly available free of charge from the KHPS website (https://www.khp.re.kr) for the purposes of academic research.

### Sample selection

**Fig. 1:**
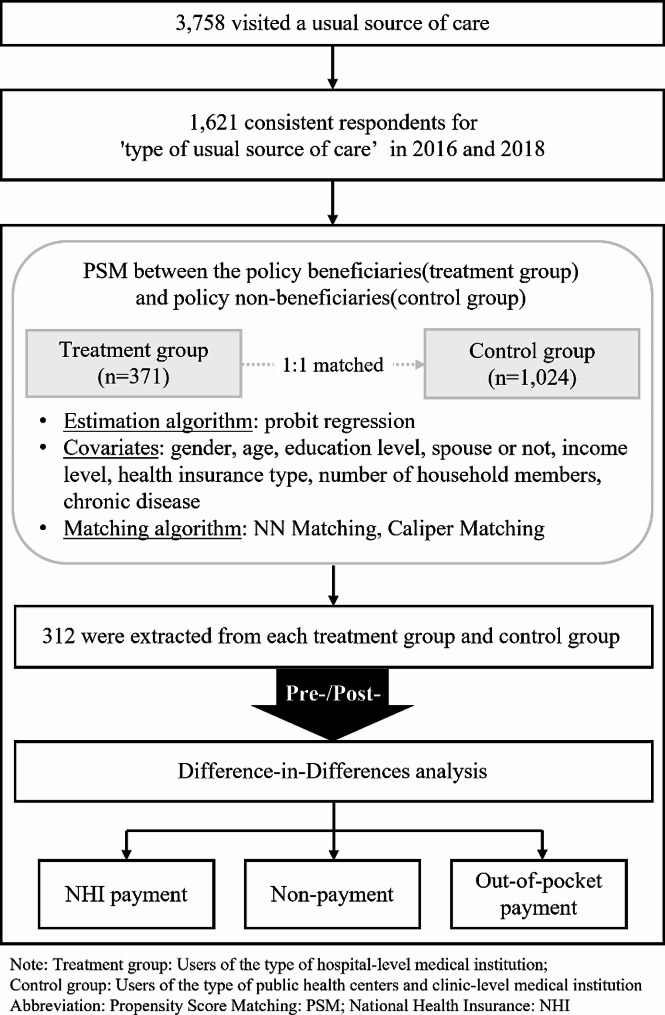
Flowchart of the study design

For this study, we selected 2016 KHP data to create a strongly balanced panel of 624 homogeneous individuals based on the following criteria. First, we selected respondents with a consistent provider to control for factors other than policy influences in the variation of an individual’s medical expenses. Specifically, 3,758 people who had medical expenses while using medical institutions were extracted from among the participants who answered “Yes” to the item “I have regular sources of care” on the KHP questionnaire. Next, to control for possible changes in healthcare utilization behavior over time, 1,621 people with consistent responses to the “Types of regular sources of care” between 2016 and 2018 were selected as participants. Subsequently, from the responses to “Types of regular sources of care,” 371 users of hospital-level or higher medical institutions were classified into the policy beneficiary group, and 1,024 users of public health centers and clinic-level medical institutions were classified under the policy as the non-beneficiary group. Finally, we used propensity score matching (PSM) to minimize the differences between the two groups, and 312 participants from the treatment and control groups were extracted, for which we performed difference-in-differences (DID) analysis (Fig. 1).

The selection of policy beneficiary groups in this study was based on the following criteria: considering the main contents of the policies applied until 2018, the policy beneficiaries are hospital-level and above. Viewing the major policies for non-payment management that were applied step-by-step until 2018 through the reinforcement measure of NHI coverage; and the abolition of optional medical expenses, health insurance coverage of the hospitalization fees for rooms meant for 2–3 people, and of the MRIs that correspond to them.

According to clauses 1 and 2 of Article 46 of the Korean Medical Law, optional medical expenses refer to those incurred when a patient or guardian selects a specific doctor to receive treatment at a hospital, general hospital, or nursing hospital. In addition, the application of health insurance to hospitalization fees for rooms meant for 2–3 people was enforced for general and high-level general hospitals, and the application of health insurance to MRI was implemented for echoencephalography, cerebral angiography, and special inspections. Clause 1 of Article 38 of the Korean Medical Law stipulates that the installation of special medical equipment, such as MRI, requires more than 200 self-owned sickbeds or the same number of shared sickbeds; thus, according to the Korean Medical Law, hospitals must have 30 or more sickbeds, and general hospitals must have 100 or more. Based on this, users of medical institutions at or above the hospital level (hospitals, general hospitals, and high-level general hospitals) were selected as the treatment and policy beneficiary groups, whereas users of public health centers and clinic-level institutions were selected as the control and policy non-beneficiary groups.

### Description of the variables

#### Dependent variable

The dependent variables for the analysis were NHI payments, OOP, and non-payments. All dependent variables were converted to natural log values for analysis due to concerns about their homoscedasticity and non-linearity. First, the NHI payments mean the amount charged and paid by the medical institution that provided the medical service to the National Health Insurance Service, only for medical services covered by the health insurance. Next, out-of-pocket expenses means the amount of medical expenses for medical services covered by health insurance minus the NHI payments. It is the amount that the patient pays for medical services covered by health insurance. Finally, non-payments mean medical expenses that are not covered by health insurance and are borne entirely by the patient [11].

#### Independent variable

We used time, grouping, interaction, and control variables as independent variables in the analysis. We coded the time variables as before-enforcement (0 = 2016) and after-enforcement (1 = 2018) based on the imposition of the Reinforcement Policy of NHI Coverage. We coded the grouping variables as the treatment group (0 = beneficiary) and the control group (1 = non-beneficiary) according to whether they benefited from the policy. We used interaction terms to identify the net effect of the policy, which we applied as the product of time and grouping variables. The control variables were selected based on predisposing, possible, and necessary factors according to the Andersen medical usage model. In terms of predisposing factors, we utilized sociodemographic factors (such as gender, age, education level, and marital status). In addition, we used income level, health insurance subscription type, and number of household members as possible factors; we used chronic diseases as necessary factors.

### Statistical analysis

#### Propensity score matching

Moon Care’s Reinforcement Plan of NHI Coverage differs in scope from year to year, which can be related to healthcare utilization behaviors such as age, income level, and disease. Therefore, the determination of the target population for the coverage enhancement policy is not entirely exogenous and may suffer from endogeneity in the self-selection process. This study aims to address this endogeneity and evaluate the net effect of reinforcement measures policy on the change in medical expenses, including non-payments. To do so, we choose a quantitative methodology based on PSM-DID, which is divided into two steps. We employed SAS 9.4 to build data for the analysis, and we examined the PSM and DID analysis using STATA 17.

First, the PSM of the following regression equation was constructed using logit regression based on a set of covariates.

$$probit\left(EY\right)={\varPhi }^{-1}\left(p\right)={\varPhi }^{-1}\left(P\left[Y=1\right]\right)={\beta }_{0}+\sum _{j=1}^{k}{\beta }_{j}{x}_{j}$$

PSM is a method of artificially constructing a control group to narrow the gap between the treatment and control groups based on the observed covariates; it is a sampling technique in which samples with similar characteristics are matched to secure homogeneity between groups [12, 13]. In this study, to ensure homogeneity between the policy beneficiaries and non-beneficiaries described above, PSM was conducted using 2016 data to form the study sample. We set whether to benefit from the Reinforcement Measure of NHI Coverage as the dependent variable, and, based on Andersen’s medical usage model, we set independent variables that can affect where to use the policy to estimate propensity scores through probit regression analysis [14]. Subsequently, based on the estimated propensity score, the sample was constructed by matching individual samples with similar scores using 1:1 NN Matching (Caliper: 0.001). NN matching is effective when the size of the control group is much larger than that of the treatment group; although it has the disadvantage that the propensity score difference between the matched samples can be large, there are fewer discarded observed values because the treatment and control groups are paired individually [15–17]. Finally, to assess the quality of the matching, we used the Balancing Test method to verify the balance of the covariates’ distributions in the treatment and control groups after matching. Furthermore, after PSM, we verified that the histograms of the propensity scores and the kernel density estimation distributions were matched. Previous studies [18–20] have shown that robustness to the quality of matching in PSMs can be performed through such balancing tests and the histogram or kernel density estimation distribution of propensity scores.

Secondly, the DID of the following regression equation was constructed based on the sample constructed through the PSM and analyzed by controlling for individual and time fixed effects.

$${Y}_{pt}={\beta }_{0}+{\beta }_{1}P+{\beta }_{2}T+{\beta }_{3}PT+\sum _{j=1}^{k}{\beta }_{j}{x}_{j}+\epsilon$$

*T* is the time variable of the treatment intervention and *P* refers to the variable of whether to benefit from the treatment. *PT* is an interaction term between the intervention time and whether to benefit from the intervention, and ${x}_{j}$ is a covariate. Therefore, the estimated volume ${\beta }_{3}$ can be calculated by estimating the net effect of the treatment on the outcome difference before and after the intervention for each group [21]. DID analysis is a quasi-experimental method used to evaluate the treatment effectiveness of policies or programs ex post facto. It divides the treatment group that benefits from the policy or program and the control group that does not, and then compares the results before and after the treatment interventions by controlling the influencing factors other than the treatment. This is an analytical technique that evaluates only the net effect of treatment through average treatment effects [22].

## Results

### Characteristics of the study population

As of 2016, the number of subjects in the treatment group before propensity score matching totaled 317, while the number of subjects in the control group totaled 1,024. After propensity score matching, the treatment and control groups were balanced at 312 each. The standardized mean difference values of the variables used in propensity score matching showed that before propensity score matching, the two groups were statistically significantly different in gender, education, income, health insurance, and chronic disease status. However, after PSM, the sociodemographic and health-related characteristics of the treatment and control groups followed a similar distribution, and the t-test and chi-square test revealed no statistically significant differences between the two groups across all variables. Hence, we can confirm that the characteristics of the variables between the treatment and control groups became similar. Regarding common characteristics between the treatment and control groups, the number of women was higher than that of men. Regarding education level, there were more high school graduates and more participants with spouses than without spouses. Furthermore, in the case of the health insurance subscription type, more job subscribers were identified and most had chronic illnesses (Table 1).

**Table 1:** General characteristics before and after PSM (2016)

		Before matching					After matching				
		Treatment group		Control group		P	Treatment group		Control group		P
		N	%	N	%		N	%	N	%	
		371	27.6	1,024	73.4		312	50.0	312	50.0	
Gender	Male	191	51.5	337	32.9	0.000^**^	141	45.2	146	46.8	0.688
	Female	180	48.5	687	67.1		171	54.8	166	53.2	
Age	Continuous (mean)	61.6		62.3		0.357	63.1		61.3		0.094
Education level	≤ Elementary school	90	24.3	348	34.0	0.000^**^	84	26.9	84	26.9	0.569
	Middle school	55	14.8	159	15.5		53	17.0	45	14.4	
	High school	125	33.7	325	31.7		114	36.5	109	35.0	
	≥ College	101	27.2	192	18.8		61	19.6	74	23.7	
Spouse or not	Yes	284	76.6	782	76.4	0.943	242	77.6	256	82.1	0.163
	No	87	23.4	242	23.6		70	22.4	56	17.9	
Income level	1st quintile (low)	58	15.6	169	16.5	0.024^*^	54	17.3	40	12.8	0.449
	2nd quintile	63	17.0	255	24.9		62	19.9	66	21.2	
	3rd quintile	84	21.0	209	20.4		73	23.4	66	21.2	
	4th quintile	89	21.5	211	20.6		62	19.9	69	22.1	
	5th quintile (high)	77	18.4	180	17.6		61	19.5	71	22.7	
Health insurance type	Employee beneficiaries	245	66.0	737	72.0	0.032^*^	209	67.0	220	70.5	0.342
	Self-employed beneficiaries	126	34.0	287	28.0		103	33.0	92	29.5	
Number of household members	Continuous (mean)	2.7		2.7		0.834	2.8		2.7		0.188
Chronic disease	Yes	353	95.2	932	91.0	0.011^*^	297	95.2	293	93.9	0.481
	No	18	4.8	92	9.0		15	4.8	19	6.1	

^*^P < 0.05, ^**^P < 0.01, propensity score matching: PSM

Chi-square tests and t-tests were performed on the matching variables in 2018 using the same subjects from 2016. Tests showed no statistically significant differences in any of the variables, with more women in both the treatment and control groups. Regarding education level, there were more high school graduates and most had a spouse. Income was evenly distributed, and there were more job subscribers in the health insurance subscription type. The number of household members was 2.7, matched equally, and most of them were found to have chronic illnesses (Table 2).

**Table 2:** General characteristics of treatment and control groups (2018)

		Treatment group		Control group		*P*
		*N*	%	*N*	%	
		312	50.0	312	50.0	
Gender	Male	141	45.2	146	46.8	0.688
	Female	171	54.8	166	53.2	
Age	Continuous (mean)	65.1		63.3		0.094
Education level	≤ Elementary school	84	26.9	83	26.6	0.647
	Middle school	52	16.7	45	14.4	
	High school	115	36.9	111	35.6	
	≥ College	61	19.5	73	23.4	
Spouse or not	Yes	241	77.2	253	81.1	0.237
	No	71	22.8	59	18.9	
Income level	1st quintile (low)	59	18.9	47	15.0	0.737
	2nd quintile	72	23.1	72	23.1	
	3rd quintile	62	19.9	63	20.2	
	4th quintile	55	17.6	57	18.3	
	5th quintile (high)	64	20.5	73	23.4	
Health insurance type	Employee beneficiaries	212	68.0	225	72.1	0.256
	Self-employed beneficiaries	100	32.0	87	27.9	
Number of household members	Continuous (mean)	2.7		2.7		0.386
Chronic disease	Yes	297	95.2	296	94.9	0.854
	No	15	4.8	16	5.1	

Propensity score matching: PSM, national health insurance: NHI

**Fig. 2:**
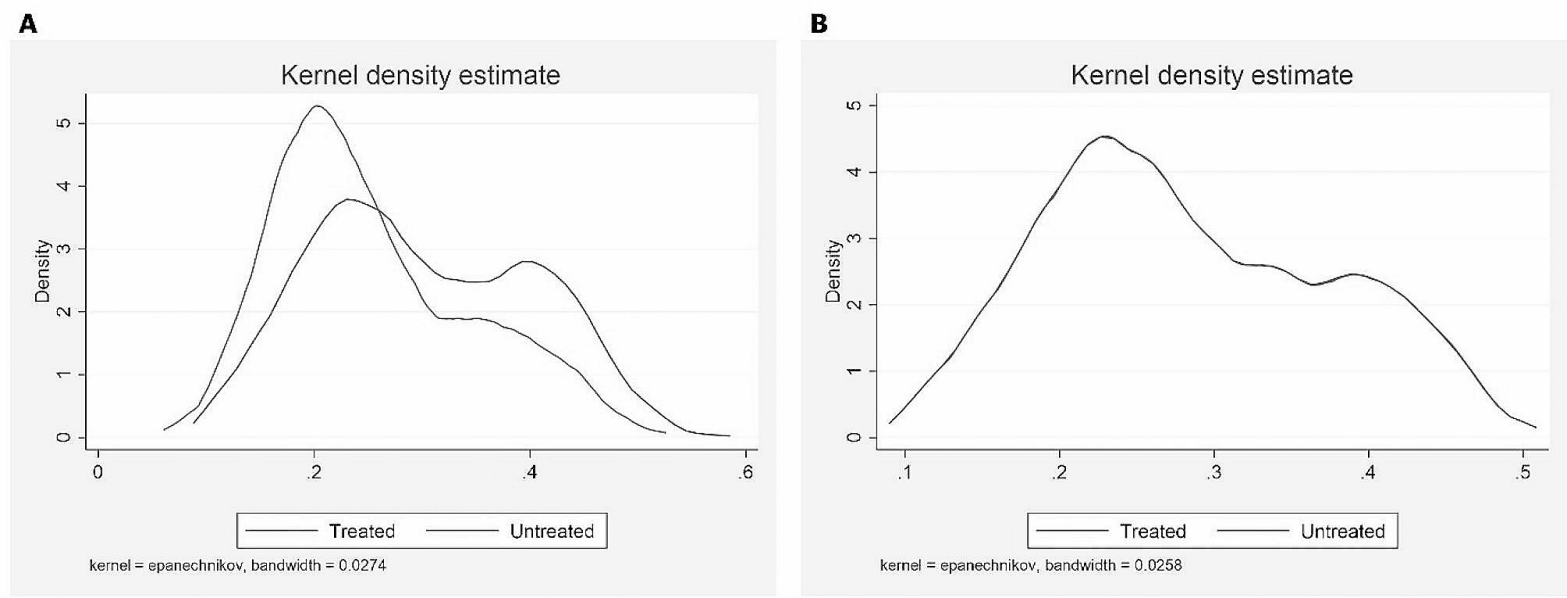
Kernel density plot before (**A**) and after (**B**) PSM

**Fig. 3:**
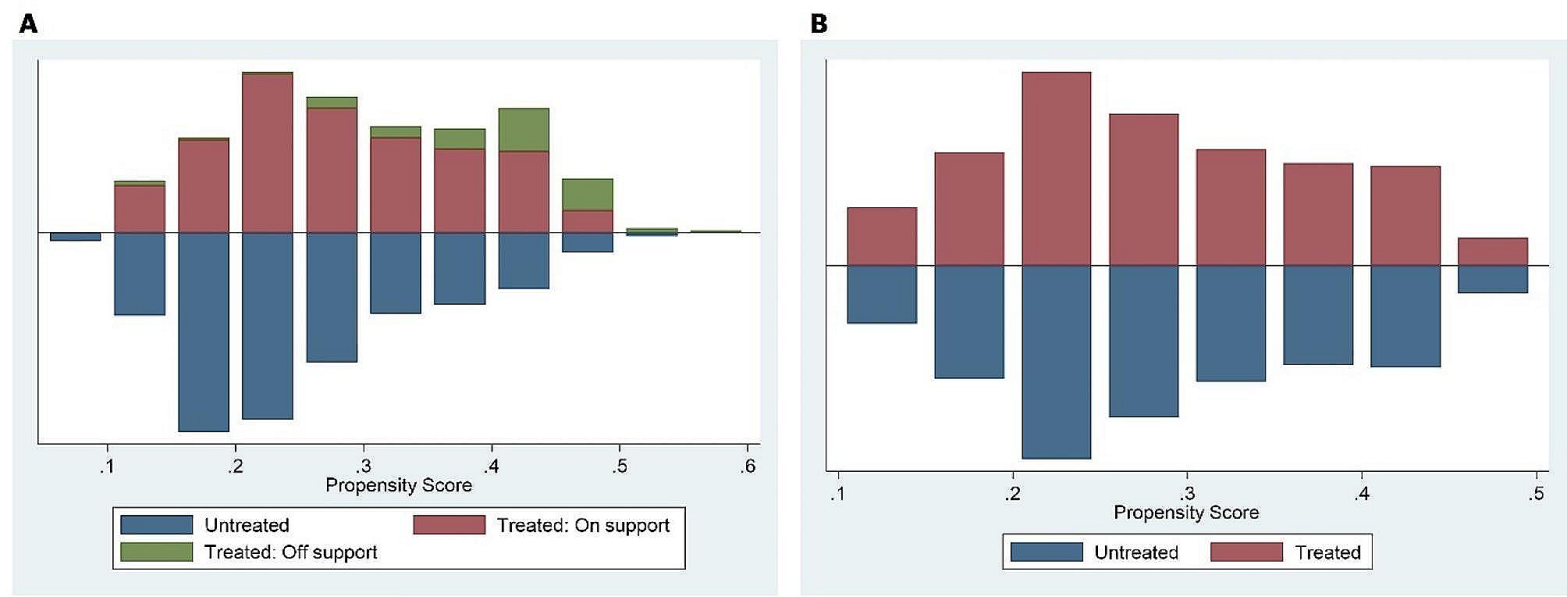
Common support graph of PSM before (**A**) and after (**B**)

The propensity score distributions of the treatment and control groups before and after PSM in 2016 and 2018 are shown in Figs. 2 and 3, respectively. By examining the propensity score kernel density plot before matching, we found that the propensity score distribution of each group was very different. The propensity score (blue solid line) of the treatment group is concentrated between 0.2 and 0.4, whereas the propensity score (red solid line) of the control group is concentrated at 0.2. However, after PSM, the kernel density distributions of the two groups were almost identical (Fig. 2). In other words, it can be seen that the PSM ensures that the beneficiaries and non-beneficiaries are similarly matched on key characteristics except for policy treatment status. The histogram distribution of the propensity scores also showed that the propensity score distributions of the two groups were similar after matching. We can see that a similar common area of propensity scores between the two groups is secured, and that matching has been properly performed based on the similarity of propensity scores (Fig. 3).

### Comparison of changes in average medical spending

Table 3 shows the difference in the average changes in medical expenses between the treatment and control groups according to the implementation of the NHI coverage reinforcement measure. For all NHI payments, non-payment items, and OOP, the control group exhibited a higher margin of increase than the treatment group. While benefiting from medical expenses after the enforcement of the policy, the treatment group (the policy beneficiary group) demonstrated a lower increase in medical expenses than the control group (the policy non-beneficiary group). As a result of two-way analysis of variance (ANOVA) comparing the average medical costs of the two groups according to policy enforcement, a statistically significant decreasing trend in non-payment items appeared after the policy was implemented. However, there were no significant differences between NHI and OOP. In the case of non-payment items, the treatment group scored higher than the control group before the policy was implemented, but scored lower than the control group after policy enforcement.

**Table 3:** Results of comparative analysis of changes in medical expenses

Outcome	Group	*N*	Before policy (2016)	After policy (2018)	Difference
NHI payment	Treatment	312	13.66	13.93	0.27
	Control	312	13.17	13.75	0.58
	Difference		0.49	0.18	-0.31
Non-payment	Treatment	312	11.96	11.53	-0.43
	Control	312	10.95	11.75	0.80
	Difference		1.01	-0.22	-1.23**
OOP	Treatment	312	13.04	13.23	0.19
	Control	312	12.22	12.71	0.49
	Difference		0.82	0.52	-0.3

^**^P < 0.01, national health insurance: NHI, out-of-pocket payments: OOP

### Results of DID analysis for measuring policy effectiveness

To identify the difference in medical expenditures between the treatment and control groups after policy enforcement, we performed a simple DID analysis by registering time variables, grouping variables, and interaction terms (Table 4). NHI payments and OOP showed statistically significant differences in the time and grouping variables, whereas the interaction term was not statistically significant. However, for non-payment items, there were statistically significant differences in all time variables, grouping variables, and interaction terms. This suggests that the non-payment costs of users of medical institutions at or above the hospital level decreased due to implementing the reinforcement measure of NHI coverage.

**Table 4:** Results of DID analysis

	NHI payment		Non-payment		OOP	
	$\beta$	*P*	$\beta$	*P*	$\beta$	*P*
Constant	13.17	0.000^**^	10.95	0.000^**^	12.22	0.000^**^
Time variable	0.58	0.001^**^	0.80	0.017^*^	0.49	0.002^**^
Grouping variable	0.48	0.007^**^	1.02	0.002^**^	0.82	0.000^**^
Interaction term	-0.30	0.252	-1.23	0.007^**^	-0.30	0.184

^*^P < 0.05, ^**^P < 0.01, difference-in-differences: DID, national health insurance: NHI, out-of-pocket payments: OOP

### Results of DID analysis for measuring policy effectiveness (with control variables)

To analyze the effect of the reinforcement measure of NHI coverage, we conducted a multiple DID analysis by registering sociodemographic and health-related variables that impact medical expenditures (Table 5). There was a statistically significant difference in the time and grouping variables in NHI payments and OOP, similar to the results of the simple DID analysis; however, there was no statistically significant difference in the interaction term, through which we could see the policy’s effectiveness. However, for non-payment items, all-time, grouping, and interaction terms were statistically significant. In particular, when considering the statistically significant decrease in the interaction term, it appears that patients’ non-payment expenditures decreased. This can be interpreted as the intervention effect of the reinforcement measure of NHI coverage that appears over time.

Specifically, the variables showing statistical significance in the dependent variables of NHI payments, non-payment items, and OOP are as follows: NHI payments were found to be greater in older age groups, but lower for those attending college or higher. Regarding non-payment items, males spent more than females; this variable was lower when males had a spouse. For OOP, it became higher as age increased, the same as NHI payments. Furthermore, the greater the number of household members, the lower the NHI payments, non-payment items, and OOP.

**Table 5:** Results of DID analysis (with control variables)

Category	Ref	NHI payment		Non-payment		OOP	
		$\beta$	*P*	$\beta$	$\beta$	*P*	$\beta$
Constant		12.74	0.000^**^	10.98	0.000^**^	11.73	0.000^**^
Time variable		0.51	0.001^**^	0.75	0.024^*^	0.44	0.004^**^
Grouping variable		0.41	0.019^*^	0.97	0.003^**^	0.76	0.000^**^
Interaction term		-0.29	0.250	-1.21	0.008^**^	-0.29	0.183
Gender	Male	0.02	0.900	0.66	0.013^*^	-0.10	0.386
Age	Continuous (Mean)	0.02	0.000^**^	0.02	0.112	0.02	0.000^**^
Education level	≤ Elementary school	-0.13	0.556	-0.09	0.789	-0.14	0.473
		-0.31	0.099	-0.28	0.366	-0.19	0.253
		-0.66	0.003^**^	-0.37	0.339	-0.45	0.021^*^
Spouse or not	Yes	-0.27	0.133	-0.78	0.021^*^	-0.13	0.418
Income level	1st quintile	-0.35	0.127	-0.31	0.400	-0.33	0.093
		-0.34	0.182	0.29	0.447	-0.20	0.346
		-0.32	0.230	0.18	0.690	-0.16	0.497
		-0.23	0.383	0.52	0.237	-0.05	0.815
Health insurance type	Employee beneficiaries	-0.13	0.344	-0.43	0.096	-0.07	0.580
Number of household members	Continuous (mean)	-0.15	0.026^*^	-0.47	0.001^**^	-0.12	0.036^*^
Chronic disease	Yes	0.03	0.918	0.19	0.787	0.17	0.586

^*^P < 0.05, ^**^P < 0.01, difference-in-differences: DID, national health insurance: NHI, out-of-pocket payments, OOP

## Discussion

This study intended to confirm whether the expansion of NHI coverage could have a positive effect on policy beneficiaries, and to verify the policy’s effect of the reinforcement measure of NHI coverage, which was enforced in 2017. Previous studies of health insurance coverage have either been limited to the four major severe diseases, assessed the relationship between national health insurance and private health insurance, or focused on out-of-pocket expenditures relative to household income. Kim and Kwon (2023) [7] & An and Kim (2020) [23] both found that catastrophic healthcare expenditure (CHE) remains an issue, particularly for lower-income groups, despite national health insurance expansions. Lee and Ko (2022) [24] noted that expanded benefits coverage reduced OOP spending without increasing healthcare utilization. Jung, Kwon, and Noh (2022) [25] emphasized that national health insurance (NHI) is more effective than PHI in reducing CHE. Finally, Lee (2022) [26] concluded that the financial burden of healthcare in Korea is regressive, with 30% of households spending over 10% of their income on medical expenses. These findings highlight the need for policy improvements to ensure equitable financial protection. Therefore, it is necessary to examine the changes before and after the introduction of the policy according to the type of medical expenses incurred by the patient’s use of medical services, which makes the evaluation of the ‘Moon Care’ policy different from the previous studies. This study differs from previous studies in that it examines the effects of the policy to expand health insurance coverage by categorizing medical expenses into three types.

As the health insurance coverage rate decreases, the responsibility for the burden of medical expenses lies more with the individual; as a disease becomes more severe, higher medical expenses are incurred, greatly affecting a household’s economic status. Accordingly, the Moon Jae-in administration tried to eliminate non-payment items that had continued since the launch of NHI in 1977 by reinforcing NHI coverage. Meanwhile, for the reinforcement measure of the coverage of the four major severe diseases, which was introduced in 2013, multilateral evaluations were made of medical costs following the introduction of the policy, whereas an evaluation has not been made of such expenses thus far in the case of the reinforcement measure of NHI coverage [27–30]. Hence, it was necessary to examine the changes in medical expenditures that appeared from people’s actual usage of medical care services five years after the policy was introduced, and then to verify the policy’s net effect.

In viewing the changes in medical expenditures of the beneficiary group after the policy was implemented, we observed that medical service fees for non-payment items significantly decreased. In addition, as a result of the DID analysis, the interaction term—through which we were able to identify the differences between groups due to policy intervention—decreased significantly, but only for non-payment items. This means that beneficiaries’ non-payment expenditures decreased after the policy was enforced, suggesting that the intervention effect of the reinforcement measure of NHI coverage over time appeared only for the non-payment items.

In sum, our results show that the policy for the reinforcement of NHI coverage, which was promoted by “covering non-payment items,” is effective. This suggests a different context from the research, finding that the expansion of NHI coverage cannot alleviate medical expenses incurred due to non-payment of medical services [7, 23, 24]. Furthermore, the results of the present study are different from the outcomes of a prior study which indicated that the outpatient OOP policy system, a medical expense support system for the elderly, will reduce a beneficiary’s OOP [31]. Non-payment items refer to medical expenses not covered by health insurance, for which the patient has to pay all medical costs, and they become a huge burden for severely ill patients. Previously implemented medical cost mitigation policies benefited only certain diseases or ages where OOP could be reduced, but they had limitations in effectively reducing non-payment expenses, the core of patients’ medical cost burden. Hence, our results underscore the effectiveness of the reinforcement measure of NHI coverage, in which existing policies were supplemented and the scope of benefits was expanded.

Although not statistically significant, the margin of increase in NHI payments and OOP was greater in the non-beneficiary group than in the beneficiary group, and their interaction terms appeared negative. This finding differs from previous results that revealed an improvement in the medical cost payment system reduces patients’ OOP [32, 33]. The reinforcement measure of NHI coverage is intended to incorporate non-payment medical services into the NHI system in the form of preliminary benefits. The preliminary benefits system was intended to minimize the balloon effect of OOP and non-payment items, which might appear as side effects of the policy. However, when viewing the outcome of not confirming a significant decrease in NHI payments and OOP, it seems that the non-payment expenses reduced by enforcing the policy did not relieve household medical costs because they were transferred to OOP or could not control for the non-payment balloon effect. Preliminary benefits refer to covering non-payment items by applying the OOP rate differentially, so an increase in OOP is inevitable, through which household health care costs will increase. Hence, there is a need for continuous discussion on NHI financial expenditures that may occur as non-payment items become incorporated into the NHI benefits system.

Meanwhile, sex, age, education level, and marital status affected medical expenditure, which is consistent with findings that women pay more than men for non-payment medical services, including X-rays and other diagnostic tests, and that age, education level, and marital status are important factors in determining the healthcare cost gap [34, 35]. In addition, the greater the number of household members, the lower the spending on NHI payments, non-payment items, and OOP. Generally, when there is a large number of household members, the share of medical expenses for each member of the household will decrease because the burden of medical expenses has been reduced, as the upper limit of household OOP for the NHI has been lowered to 10% of annual income due to the reinforcement measure of NHI coverage. In other words, given that medical spending changes according to sociodemographic characteristics, it is necessary to complement the policy by setting indicators for each subgroup to increase the future NHI coverage rate.

This study has several limitations. First, sufficient time was not provided to measure the policy’s effect. The reinforcement measure of NHI coverage was enforced for five years, and it is appropriate to utilize all data for the policy implementation period; however, we examined the policy’s effect only for 2018 owing to limitations in the data. Second, the study does not account for unobservable heterogeneity. While we ensured the homogeneity of the beneficiaries and non-beneficiaries before comparing changes in healthcare costs due to policy implementation, this study was not an experimental study and we were unable to control for unobservable characteristics (risk attitudes, personality, etc.) that may affect healthcare cost changes. Third, the comparative groups could not be further subdivided. For participants already receiving benefits from the medical expense relief policy, such as those with the four major severe diseases or recipients of medical benefits, this policy’s effect may be insignificant. To prepare detailed policies for each group in the future, it will be necessary to include patients with severe diseases or recipients of medical benefits as a comparative group for analysis. Fourth, we did not consider patients with private health insurance. In South Korea, where there are many non-payment items, private health insurance is intended to relieve the burden of non-payment items; however, in the event of a low-income bracket, there is a limit to private insurance subscriptions. In future research, it will be necessary to analyze changes in private health insurance according to the enforcement of policies in order to determine whether the burden of medical expenses has been alleviated.

## Conclusion

Despite the limitations mentioned above, this study is the first, to the best of our knowledge, to examine changes in national medical expenses according to the reinforcement measure of NHI coverage, which has been enforced since 2017, and to verify the policy’s effectiveness. The policy’s positive effect was confirmed as the non-payment costs of policy beneficiaries decreased. However, there were limitations in identifying the mitigating effects of NHI and OOP. To overcome this and ease the burden of national medical expenses by expanding the health insurance coverage rate, the following policy implications are suggested.

First, it is necessary to expand the health insurance coverage rate by continuously promoting coverage of non-payment items. Medical practices, including ultrasonography and MRI, which are essential for disease diagnosis but belong to non-payment categories, will reduce accessibility to medical care due to their high exam costs. In particular, they place a great burden on vulnerable people (such as low-income individuals and the older people), acting as a factor in causing medical blind spots. If the health insurance coverage rate is expanded to cover non-payment items, high-quality medical services can be provided to all people, preventing blind spots and meeting their medical needs.

Second, it is necessary to secure the fiscal soundness of health insurance by managing excessive medical care users owing to the expansion of benefits. The use of a preliminary benefits system to expand the NHI coverage rate can lead to health insurance financial burdens by increasing the NHI and OOP. However, in the long term, strengthening medical accessibility by expanding the NHI coverage rate can enable the early detection of diseases and ultimately reduce national medical expenses through disease prevention. It is necessary to introduce a family doctor system to manage the excessive medical care that may occur in the short term. Thus, overdiagnosis and excessive outpatient use for patients with chronic illnesses can be prevented, and the use of tertiary hospitals can be suppressed by establishing a primary medical system.

Third, subdivided indicators must be prepared for each population subgroup to measure the NHI coverage rate. The NHI coverage rate currently used in the policy is measured based on the entire population and hence does not reflect coverage rates that may appear differently depending on the subgroup. As the NHI coverage rate experienced by people differs according to age, income level, and disease status, it is necessary to measure the NHI coverage rate using diverse indicators such as impoverishment, un-attended medical care needs, and catastrophic medical expenses. Thus, it will be possible to supplement policies effectively by subdividing them into policy target groups, income brackets, and diseases.

## Data Availability

The tools and resources outlined in this manuscript are publicly available from the Korean Health Panel website (https://www.khp.re.kr:444/).

## Abbreviations

NHI: National Health Insurance
UHC: Universal Health Coverage
GPD: Gross Domestic Product
OECD: Organization for Economic Co-operation and Development
OOP: Out-of-Pocket Payments
KHP: Korean Health Panel
MRI: Magnetic Resonance Imaging
PSM: Propensity Score Matching
DID: Difference In Differences

## References

[1] Wagstaff A, Neelsen S (2020). A comprehensive assessment of universal health coverage in 111 countries: a retrospective observational study. Lancet Global Health.

[2] Campbell T, Galvani AP, Friedman G, Fitzpatrick MC (2022). Exacerbation of COVID-19 mortality by the fragmented United States healthcare system: a retrospective observational study. Lancet Reg Health-Americas.

[3] Lee DW, Jang J, Choi D-W, Jang S-I, Park E-C (2020). The effect of shifting medical coverage from National Health Insurance to Medical Aid type I and type II on health care utilization and out-of-pocket spending in South Korea. BMC Health Serv Res.

[4] Huh SI (2021). Analysis of the National Health Insurance Coverage Expansion Policy after single-payer system. Korean Association Social Policy.

[5] Kim S (2020). Changes in Household Health expenditure after Health Insurance Coverage Expansion and their policy implication. Health Welf Policy Forum.

[6] Park E-C (2017). Moon Jae-in Government’s plan for benefit expansion in National Health Insurance. Health Policy Manage.

[7] Kim S, Kwon S. Has South Korea achieved the goals of national health insurance? Trends in financial protection of households between 2011 and 2018. Soc Sci Med 2023:115929. 10.1016/j.socscimed.2023.115929 37137200

[8] Park E-C (2019). Direction of healthcare reform for sustainability. Health Policy Manage.

[9] Lee Y, Kim S, Kim SY, Kim G (2019). Ethical consideration of National Health Insurance Reform for universal health coverage in the Republic of Korea. Asian Bioeth Rev.

[10] Park S, Lee K-S, Choi M, Lee M (2022). Factors associated with quality of life in patients with benign prostatic hyperplasia, 2009–2016. Medicine.

[11] Ku Y-C, Chou Y-J, Lee M-C, Pu C (2019). Effects of National Health Insurance on household out-of-pocket expenditure structure. Soc Sci Med.

[12] Cousineau M, Verter V, Murphy SA, Pineau J. Estimating causal effects with optimization-based methods: a review and empirical comparison. Eur J Oper Res 2022. 10.1016/j.ejor.2022.01.046 PMC12657020 41311879

[13] Bertoni D, Curzi D, Aletti G, Olper A (2020). Estimating the effects of agri-environmental measures using difference-in-difference coarsened exact matching. Food Policy.

[14] Niknam BA, Zubizarreta JR (2022). Using cardinality matching to design balanced and representative samples for observational studies. JAMA.

[15] Wrubel E, Natwick R, Wright GP (2021). Breast-conserving therapy is associated with improved survival compared with mastectomy for early-stage breast cancer: a propensity score matched comparison using the national cancer database. Ann Surg Oncol.

[16] Fortin S, Johnston S, Coplan P, Zubizarreta J (2020). PMD49 COMPARISON OF CARDINALITY MATCHING VS PROPENSITY SCORE MATCHING FOR TARGETED ESTIMANDS. Value Health.

[17] Evans SZ (2021). Propensity score matching. Encyclopedia Res Methods Criminol Criminal Justice.

[18] Zhou Q, He Q, Eggleston K, Liu GG (2022). Urban-rural health insurance integration in China: impact on health care utilization, financial risk protection, and health status. Appl Econ.

[19] Li C, Tang C, Wang H (2019). Effects of health insurance integration on health care utilization and its equity among the mid-aged and elderly: evidence from China. Int J Equity Health.

[20] Yang S, Guo D, Bi S, Chen Y (2023). The effect of long-term care insurance on healthcare utilization of middle-aged and older adults: evidence from China health and retirement longitudinal study. Int J Equity Health.

[21] Wing C, Simon K, Bello-Gomez RA (2018). Designing difference in difference studies: best practices for public health policy research. Annu Rev Public Health.

[22] Perraillon MC, Lindrooth R, Welton JM (2019). Difference-in-difference research designs. Nurs Econ.

[23] An J, Kim S (2020). Medical cost trends under national health insurance benefit extension in Republic of Korea. Int J Health Plann Manag.

[24] Lee HM, Ko H (2022). The impact of benefits coverage expansion of social health insurance: evidence from Korea. Health Policy.

[25] Jung HW, Kwon YD, Noh J-W (2022). How public and private health insurance coverage mitigates catastrophic health expenditures in Republic of Korea. BMC Health Serv Res.

[26] Lee C (2022). Is Universal Health Insurance Superior in Terms of Healthcare payment? Estimating Financial Burden of Healthcare in Korea: 2009 to 2019. INQUIRY: J Health Care Organ Provis Financing.

[27] Lee H-Y, Oh J, Kawachi I (2022). Changes in Catastrophic Health expenditures for Major diseases after a 2013 Health Insurance Expansion in South Korea: study examines changes in catastrophic health expenditures in South Korea. Health Aff.

[28] Jung H, Lee J (2021). Estimating the effectiveness of national health insurance in covering catastrophic health expenditure: evidence from South Korea. PLoS ONE.

[29] Kim S, Kwon S (2015). Impact of the policy of expanding benefit coverage for cancer patients on catastrophic health expenditure across different income groups in South Korea. Soc Sci Med.

[30] Lee M, Yoon K (2019). Catastrophic health expenditures and its inequality in households with cancer patients: a panel study. Processes.

[31] Han S, Sohn H. The short-term effects of fixed copayment policy on elderly health spending and service utilization: evidence from South Korea’s age-based policy using exact date of birth. Int J Health Econ Manage 2023:1–25. 10.1007/s10754-023-09344-1 36849754

[32] Jian W, Lu M, Chan KY, Poon AN, Han W, Hu M, Yip W (2015). Payment reform pilot in Beijing hospitals reduced expenditures and out-of-pocket payments per admission. Health Aff.

[33] Lee G, Lee J (2020). The impact of the policy of expanding coverage for 4 major diseases on out-of-pocket payments. Eur J Pub Health.

[34] Mondal B, Dubey JD (2020). Gender discrimination in health-care expenditure: an analysis across the age-groups with special focus on the elderly. Soc Sci Med.

[35] Brinda EM, Kowal P, Attermann J, Enemark U (2015). Health service use, out-of-pocket payments and catastrophic health expenditure among older people in India: the WHO study on global AGEing and adult health (SAGE). J Epidemiol Community Health.

